# Reproductive Ageing: Inflammation, immune cells, and cellular senescence in the aging ovary

**DOI:** 10.1530/REP-23-0499

**Published:** 2024-06-21

**Authors:** José V V Isola, Jessica D Hense, César A P Osório, Subhasri Biswas, José Alberola-Ila, Sarah R Ocañas, Augusto Schneider, Michael B Stout

**Affiliations:** 1 Aging & Metabolism Research Program, Oklahoma Medical Research Foundation, Oklahoma City, Oklahoma, USA; 2 Nutrition College, Universidade Federal de Pelotas, Pelotas, RS, Brazil; 3 Arthritis & Clinical Immunology Research Program, Oklahoma Medical Research Foundation, Oklahoma City, Oklahoma, USA; 4 Genes & Human Disease Research Program, Oklahoma Medical Research Foundation, Oklahoma City, Oklahoma, USA; 5 Oklahoma City Veterans Affairs Medical Center, Oklahoma City, Oklahoma, USA

## Abstract

**In brief:**

Recent reports suggest a relationship between ovarian inflammation and functional declines, although it remains unresolved if ovarian inflammation is the cause or consequence of ovarian aging. In this review, we compile the available literature in this area and point to several current knowledge gaps that should be addressed through future studies.

**Abstract:**

Ovarian aging results in reduced fertility, disrupted endocrine signaling, and an increased burden of chronic diseases. The factors contributing to the natural decline of ovarian follicles throughout reproductive life are not fully understood. Nevertheless, local inflammation may play an important role in driving ovarian aging. Inflammation progressively rises in aged ovaries during the reproductive window, potentially affecting fertility. In addition to inflammatory markers, recent studies show an accumulation of specific immune cell populations in aging ovaries, particularly lymphocytes. Other hallmarks of the aging ovary include the formation and accumulation of multinucleated giant cells, increased collagen deposition, and increased markers of cellular senescence. Collectively, these changes significantly impact the quantity and quality of ovarian follicles and oocytes. This review explores recent literature on the alterations associated with inflammation, fibrosis, cell senescence, and the accumulation of immune cells in the aging ovary.

## Introduction: A brief overview of the ovarian life cycle

In humans, the ovarian follicles are established during early fetal development and completed before birth (Bendsen *et al.* 2006). During fetal organogenesis, peak female gamete development occurs in the second trimester. By the 20th week of gestation, the newly developed ovaries have as many as seven million quiescent primordial follicles (PF) arrested in meiotic prophase I with diminished transcriptional and translational activity. However, at birth, only one to two million PFs remain due to atresia-mediated declines (Block 1951, Baker 1963). At the time of menarche, only 300,000 to 500,000 PFs remain in human ovaries (Kelsey *et al.* 2011). A similar pattern occurs in mice, except that the PF pool is not fully established until shortly after birth, usually within the first 3 days postnatally (Kerr *et al.* 2013). In the first week of life, mice possess approximately 10,000 PFs (Bristol-Gould *et al.* 2006), although at least half of these undergo atresia before sexual maturation (Tingen *et al.* 2009).

The female reproductive life span typically refers to the time from sexual maturity until the onset of reproductive failure. Declines in fecundity result from interrelated intrinsic and extrinsic factors, several of which occur in the ovary itself (Yureneva *et al.* 2021). Similar to other organ systems, the ovary presents age-related changes in morphology and endocrine function, which are mirrored by changes in cellularity (number and type of cells) and architecture (Amanvermez & Tosun 2016, Isola *et al.* 2024). During the reproductive period, the ovarian reserve declines dramatically in humans and mice. The ovarian reserve consists primarily of PFs, and to a lesser extent, growing follicles including primary, secondary, tertiary (antral), and preovulatory (Graafian) follicles (Gleicher *et al.* 2011). In humans, the loss of PFs remains relatively constant at approximately 1000 follicles per month up until 37 years of age, when the rate of decline accelerates until the onset of menopause (Broekmans *et al.* 2007, Park *et al.* 2022). In most strains of mice, the number of PFs decreases by over half from 3 to 12 months of age, with a significant drop occurring between 6 and 9 months of age (Kevenaar *et al.* 2006, Pelosi *et al.* 2013, Ansere *et al.* 2021, Zhou *et al.* 2022). In most strains, 9–12 months of age represents the period when mice begin experiencing irregular estrous cycles (Koebele & Bimonte-Nelson 2016). After 12 months of age, the PF pool continues to decline at a decelerated rate, and estrus cycling ultimately arrests (estropause) around 15–16 months of age (Maffucci & Gore 2006).

The changing ovarian microenvironment during aging has ramifications beyond reducing follicle numbers. In particular, oocyte quality is reduced with advancing age (Nagamatsu 2023), which adversely affects potential embryo viability and pregnancy rates (Broekmans *et al.* 2009, Franasiak *et al.* 2014). Oocyte health is closely linked to redox homeostasis (Yan *et al.* 2022, Shi *et al.* 2023). With advancing age, antioxidant defenses decrease in the ovary, which contributes to oocyte DNA damage, telomere shortening, and mitochondrial dysfunction, all of which have been reviewed extensively in prior reports (Camaioni *et al.* 2022, Yan *et al.* 2022, Shi *et al.* 2023). In brief, oocyte reactive oxygen species (ROS) promote DNA double-strand breaks (DSBs) and telomere attrition by oxidizing guanine-rich telomeric DNA (Yamada-Fukunaga *et al.* 2013). The concomitant rise in ROS and decreased antioxidant defenses synergistically drive ovarian functional declines and infertility (Lim & Luderer 2011, King *et al.* 2023). However, changes in oocyte redox homeostasis do not occur independently. Age-related pro-inflammatory processes are activated in parallel with ovarian oxidative stress and mitochondrial dysfunction (Ansere *et al.* 2021, Umehara *et al.* 2022, Isola *et al.* 2024). Although these recent reports suggest a relationship between ovarian inflammation and functional declines, it remains unresolved if ovarian inflammation is a cause or consequence of ovarian aging. We speculate that ovarian inflammation and immune cell accumulation are causally implicated in age-related ovarian failure because they precede infertility and are associated with stromal fibrogenesis and multinucleated giant cell (MNGC) accumulation in mice (Briley *et al.* 2016). In the following sections, we concisely review the available literature in this area and focus on several knowledge gaps at present that should be addressed through future studies.

## Age-related pro-inflammatory stress in the ovary

Aging is marked by a progressive and sustained increase in systemic pro-inflammatory stress (Cesari *et al.* 2004, Stout *et al.* 2017). Age-related inflammation often occurs independently of infection (i.e. sterile) and is commonly referred to as ‘inflammaging’ (Franceschi *et al.* 2000, Di Giosia *et al.* 2022). In mice and humans, circulating pro-inflammatory cytokines linked to inflammaging include interleukin (IL)-6, IL-1, IL-8, IL-18 and tumor necrosis factor-α (TNF-α), which are also associated with age-related diseases (Franceschi & Campisi 2014). Inflammatory processes play a natural role in regulating ovarian remodeling associated with follicular atresia, ovulation, and regression of the corpus luteum (CL) (Duffy *et al.* 2019). However, aging exacerbates ovarian pro-inflammatory responses well before reproductive failure (Briley *et al.* 2016, Zhang *et al.* 2020, Lliberos *et al.* 2021, Umehara *et al.* 2022, Isola *et al.* 2024). At the transcriptional level, many of the age-related changes in the ovary are related to inflammation and immune-related responses, as evidenced by upregulation of antigen processing/presentation, innate immune activation responses, leukocyte adhesion and signaling, IL-8 signaling, IL-17 signaling, S100 family signaling, and toll-like receptor activation pathways (Sharov *et al.* 2008, Schneider *et al.* 2017, Ma *et al.* 2020, Zhang *et al.* 2020, Ansere *et al.* 2021, Isola *et al.* 2024). Pro-inflammatory cytokine (IL-6, IL-1β, IL-8, interferon-gamma (INF-γ), TNF-α) and chemokine (monocyte chemoattractant protein-1 (*Ccl2*), C-C motif chemokine ligand (*Ccl*) 5, and C-X-C motif chemokine ligand (*Cxcl*) 2) levels also increase within the aged ovary (Briley *et al.* 2016, Lliberos *et al.* 2021, Umehara *et al.* 2022). A recent proteomics study in murine ovary also demonstrated an age-related increase in several markers of pro-inflammatory stress and immune responses, suggesting increased chemotaxis and lymphocyte-mediated immunity (Dipali *et al.* 2023). Although the contributions of individual ovarian cell types and immune cells to these phenotypic changes remain unclear, the overall changes are aligned with what is reported in a recent single-cell transcriptomic and fluorescence-activated cell sorting analyses (Isola *et al.* 2024). Specifically, pro-inflammatory transcriptomic changes occur in granulosa and theca cells before reproductive failure, suggesting that the follicles contribute to the pro-inflammatory microenvironment in the aging ovary (Duncan *et al.* 2017). Human studies have revealed similar findings to the mouse data outlined above. For instance, IL-3, IL-6, IL-7, IL-15, CCL3, and CXCL10 are reported to increase in follicular fluid from women over the age of 38 years (McReynolds *et al.* 2012, Hashemitabar *et al.* 2014, Machlin *et al.* 2021). These studies indicate that age-related increases in ovarian pro-inflammatory stress are conserved across species and may be causally linked to reductions in oocyte quality, difficulties with conception, and the emergence of reproductive failure (Broekmans *et al.* 2009).

The rise in pro-inflammatory pathways in the aged ovary is also paralleled by reparative responses that can become maladaptive. For instance, transforming growth factor-β (TGF-β) signaling is important for theca and granulosa cell communication during follicular growth (Knight & Glister 2006) and also provoked during aging. Enhanced TGF-β activity has been linked to lymphocyte chemotaxis and activation (Li *et al.* 2006, McCloskey *et al.* 2020), which increases in the aging ovary (Dipali *et al.* 2023, Isola *et al.* 2024). Moreover, TGF-β activity plays a well-established role in driving collagen accumulation and fibrosis during aging (Ren *et al.* 2023). Notably, collagen accumulation is a hallmark of ovarian aging and has been reported in both mice (Briley *et al.* 2016, Umehara *et al.* 2022, Isola *et al.* 2024) and humans (Amargant *et al.* 2020, McCloskey *et al.* 2020). Interestingly, the expression of ovarian collagen genes seems to remain stable with age during the reproductive window (Lliberos *et al.* 2021, Isola *et al.* 2024). However, collagenase pathway activity decreases in the aged ovary (Isola *et al.* 2024), likely contributing to age-related ovarian fibrosis and impairments in ovulation (Umehara *et al.* 2022). Age-related ovarian fibrosis is also closely associated with proteomic changes indicative of a pro-inflammatory environment within the ovary (Dipali *et al.* 2023). Interestingly, pharmacological interventions (e.g. metformin, BGP-15 [(O-[3-piperidino-2-hydroxy-1-propyl]-nicotinic amidoxime)]) that attenuate ovarian fibrosis also curtail ovarian pro-inflammatory stress (McCloskey *et al.* 2020, Landry *et al.* 2022, Umehara *et al.* 2022), further supporting their interactive role in accelerating ovarian aging. *Il4* and *Il13*, which are generally considered anti-inflammatory cytokines, are also increased in the aged ovary (Zhang *et al.* 2020, Umehara *et al.* 2022). These cytokines promote macrophage polarization toward an alternatively activated phenotype (Zhang *et al.* 2020), which is thought to serve an important role in ovarian remodeling (Umehara *et al.* 2022). Notably, alternative activation of macrophages has also been linked to the formation and accumulation of MNGCs (Martinez *et al.* 2009), which increase in the aged ovary and may play a deleterious role in ovarian function (Asano 2012, Briley *et al.* 2016, Foley *et al.* 2021, Isola *et al.* 2024).

Based on the data described above, suppressing pro-inflammatory pathways may extend the reproductive window and delay ovarian aging. Indeed, the global ablation of IL-1α in mice decreased ovarian *Il1b*, *Tnfa*, and *Il6*, which was associated with increased ovarian reserve and improved fertility (Uri-Belapolsky *et al.* 2014). Similarly, the ablation of the NLRP3 inflammasome also increased the ovarian reserve and circulating anti-Müllerian hormone levels in 12-month-old mice, leading to greater pregnancy rates and litter sizes than those observed in age-matched controls (Navarro-Pando *et al.* 2021). The NLRP3 inflammasome is one of the most extensively characterized endogenous danger signals in mammals, and its role in inflammaging is well established (Cordero *et al.* 2018, Latz & Duewell 2018). Despite these observations, it remains unclear if the suppression of ovarian pro-inflammatory responses in global IL-1α and NLRP3 knockout mice occurs due to direct ablation in ovarian cell types or in response to systemic declines in pro-inflammatory signaling pathways. To our knowledge, no studies have directly ablated pro-inflammatory mediators in ovarian cell types. Thus, this represents an area of study that merits additional investigation.

## Age-related immune cell function and accumulation in the ovary

Inflammatory processes play a natural role in regulating folliculogenesis and ovarian remodeling (Duffy *et al.* 2019). Therefore, immune cells in the ovary are essential for normal ovarian function. An overview of the general roles of immune cells, their physiological function in the ovary, and potential outcomes in ovarian aging is presented in Table 1. In particular, macrophages play a critical role in follicle growth, atresia, ovulation, and CL formation and regression (Gaytan *et al.* 1998, Wu *et al.* 2004, Zhang *et al.* 2021). For instance, macrophages recognize and phagocytose apoptotic and necrotic cells as well as cellular debris. Therefore, they are crucial for the removal of atretic granulosa cells and apoptotic luteal cells (Gaytan *et al.* 1998). Macrophages also promote follicular growth and survival by secreting growth factors that stimulate cell proliferation and suppress follicular apoptosis (Fukumatsu *et al.* 1992, Tingen *et al.* 2011, Ono *et al.* 2018). During ovulation, macrophages infiltrate periovulatory follicles and produce cytokines important for rupturing the follicle (Van der Hoek *et al.* 2000, Zhang *et al.* 2021). After ovulation, macrophages regulate CL development by supporting the microvascular network required for CL integrity and production of progesterone that is essential for establishing pregnancy (Care *et al.* 2013). Other studies suggest that macrophages support vascular integrity through direct interaction and cross talk with ovarian endothelial cells (Turner *et al.* 2011, Li *et al.* 2022). Distinct populations of ovarian macrophages, including resident and monocyte-derived, have been identified (Zhang *et al.* 2020, Li *et al.* 2022, Zhou *et al.* 2023), but the precise role that each population plays and how phenotype switching may influence adaptive and maladaptive functions remains unclear.

**Table 1:** Studies reporting on the role of immune cells in ovarian pathology.

Immune cell subsets	Physiological function	Functional significance in the ovary	Reference
Double-negative innate T cells (CD4− CD8−)	Lymphoid cells with rearranged antigen receptors that bridge innate and adaptive immunity; respond rapidly to activation through their TCRs or by cytokines and influence early immune responses.	Support steroidogenesis; CL maintenance and regression	Poole & Pate (2012), Walusimbi & Pate (2013), Bafor *et al.* (2022)
Type 1 (αβ DN, NKT, MAIT, γδ DN)			
Type 17 (αβ DN, NKT, MAIT, γδ DN)			
Conventional αβ T cells (TC)			
CD8+ T cells (cytotoxic)	Respond to diverse antigens presented by MHC class I molecules by proliferating, secreting cytokines and chemokines, and directly lysing infected cells	Aid in ovulatory process; cell-mediated inflammatory response in regression of CL	Bukulmez & Arici (2000)
Conventional CD4+ T cells (helper)	Aid the activity of other immune cells by releasing cytokines; essential in B cell antibody class switching, activation and growth of cytotoxic T cells, and maximizing bactericidal activity of phagocytes	Secrete cytokines (interleukins, TNF-α, IFN-γ) crucial for ovulatory process; luteal prostaglandin production; luteal regression	Pate (1995), Knapik *et al.* (2022)
CD4+ FOXP3+ Tregs	A specialized subpopulation of T cells that acts to suppress immune response, thereby maintaining homeostasis and self-tolerance; express high levels of FOXP3	Tregs in the ovaries are potent suppressors of autoimmunity and play a crucial role in the tolerance of allogeneic pregnancy-related tissues and autologous oocytes	Knapik *et al.* (2022)
Innate lymphoid cells (ILC1, ILC2, ILC3)	Tissue resident cells. Involved in early responses to pathogens, as well as in tissue repair after injury.	May orchestrate other cell types participating in cycles of developing and regressing structures in the ovary	Ben Yaakov *et al.* (2023)
B cells	T cell-dependent immune response to foreign antigens by antibody secreting plasma cells and memory B cell; T cell-independent response to blood borne pathogens	Remains underexplored	
Natural killer cells	Highly efficient anti-tumor effectors; kill target cells without previous sensitization	Co-infiltrate with cytotoxic T cells and are strongly associated with ovarian cancer tumor infiltration	Nersesian *et al.* (2019)
Dendritic cells	Professional antigen-presenting cells that capture, process, and present antigens to lymphocytes to initiate and regulate the adaptive immune response	Upregulates specific ovulatory genes crucial for cumulus mucification/expansion and ovulation; facilitates progesterone production and ovarian lymphangiogenesis in the newly formed CL	Cohen-Fredarow *et al.* (2014)
Monocytes	Contribute to the initiation, development and resolution of inflammation; tissue regeneration and repair processes; promoting angiogenesis and vascular remodeling	Participates in maintenance of ovarian follicle health, ovarian follicle maturation in preparation for ovulation, CL formation and regression	Younesi *et al.* (2022)
Macrophages (Mø)	While classically activated macrophages (M1 Mø) protect the host from a variety of bacteria, protozoa, and viruses and play critical role in antitumor immunity, alternatively activated macrophages (M2 Mø) have tissue remodeling and wound healing function	Promotes follicular growth and survival by stimulating cell proliferation and suppressing follicular apoptosis; infiltrate periovulatory follicles and produce cytokines required for follicle rupture; regulate CL development by supporting vascular integrity and progesterone production; removal of atretic granulosa cells and apoptotic luteal cells	Fukumatsu *et al.* (1992), Gaytan *et al.* (1998), Van der Hoek *et al.* (2000), Tingen *et al.* (2011), Care *et al.* (2013), Ono *et al.* (2018), Zhang *et al.* (2021)
Granulocytes			
Neutrophils	Primary mediators of rapid innate host defense against most bacterial and fungal pathogens; capture and destroy invading microorganisms, through phagocytosis, intracellular degradation, and release of granules	Aid in functional (decreased progesterone) and structural (cell death) regression of the corpus luteum; induces capillary-like structures in luteal endothelial cells indicating a role in luteal angiogenesis	Murdoch & Steadman (1991), Jiemtaweeboon *et al.* (2011), Walusimbi & Pate (2013)
Eosinophils	Maintenance of homeostasis; host defense against infectious agents; innate immunity activities; immune regulation through Th1/Th2 balance, anti-inflammatory, and anti-tumorigenic effects recruitment and homeostasis	Have a role in the mechanics of ovulation and luteal regression. Eosinophil influx occurs during the period of angiogenesis and luteinization, supporting the proposal that eosinophils may influence both events	Reibiger & Spanel-Borowski (2000), Walusimbi & Pate (2013)

CL, corpus luteum; FOXP3, forkhead box P3; TCRs, T-cell receptors; Tregs, regulatory T cells.

The effect of aging on macrophage accumulation in the ovary remains unresolved. While some reports indicate that macrophage numbers increase in aged ovaries (Lliberos *et al.* 2021, Umehara *et al.* 2022), others report no changes (Briley *et al.* 2016, Isola *et al.* 2024). Further complicating this issue, other reports indicate that the overall number of ovarian macrophages is actually reduced with age (Zhang *et al.* 2020, Ben Yaakov *et al.* 2023) (Table 2). The reasons underlying these discrepancies are unresolved, but differences in methodological approaches, age when mice were evaluated, strain, and cycle stage may contribute to the reported differences across studies. Another potential contributor may be the incorporation of macrophages into MNGCs within the aged ovary. However, a few inferences can be ascertained from the collective body of literature. For instance, resident ovarian macrophages appear to decline with aging in both mice and humans, while monocyte-derived macrophages likely increase (Zhang *et al.* 2020, Zhou *et al.* 2023). Another consistent observation is that aging appears to shift ovarian macrophages toward an alternatively activated state, which is postulated to accumulate in response to constant remodeling over time (Zhang *et al.* 2020, Umehara *et al.* 2022). Interestingly, a higher proportion of alternatively activated ovarian macrophages has also been observed in mice with ovarian fibrosis (McCloskey *et al.* 2020), suggesting they are at least a biomarker of ovarian stress if not causally implicated in ovarian aging. As alluded to above, alternatively activated macrophages likely contribute to the formation of MNGCs (Martinez *et al.* 2009), which increase significantly in the aged ovary (Asano 2012, Briley *et al.* 2016, Foley *et al.* 2021, Isola *et al.* 2024). Notably, prior reports suggest that MNGCs are at least partially composed of macrophages due to F4/80 immunoreactivity (Briley *et al.* 2016), a surface marker of mouse macrophages (Murray & Wynn 2011). The functional importance of MNGCs remains unclear but they possess high phagocytic capacity (Milde *et al.* 2015), which may serve a role in resolving fibrotic regions within the ovary. Additional studies are needed to elucidate what role MNGCs serve in the ovary and if they are pathogenetic or compensatory.

**Table 2:** Studies reporting on age-related outcomes in immune cells and ovarian pathology.

Age-related outcomes in immune cells	Implications in ovarian aging	Methodological approach	Reference
Double-negative innate T cells (CD4^−^ CD8^−^)			
Type 1 NKT cells ↑; type 1 αβ DN and γδ DN ↓	Elevated type 1 NKTs might provide immune responses against fibrosis and inflammation induced in aging ovary	Single-cell RNA seq; flow-cytometric analysis	Isola *et al.* (2024)
Type 17 αβ DN, NKT, γδ DN, MAIT↑	Increased type 17 γδ DN could either promote fibrosis or be a compensatory attempt to dampen the fibrotic environment	Single-cell RNA seq; flow-cytometric analysis	Ben Yaakov *et al.* (2023), Isola *et al.* (2024)
Conventional αβ T cells (TC)			
CD8+ T cells (cytotoxic): no significant change or modestly increase	Down regulation of CD4 and CD8 due to chronic stimulation can give rise to DN population	Single-cell RNA-seq; flow-cytometric analysis	Lliberos *et al.* (2021), Ben Yaakov *et al.* (2023), Isola *et al.* (2024)
CD4+ T cells ↑ or no significant change	Might cause pro-inflammatory changes in ovarian microenvironment. If decreased, might hinder the ability to mount an immune response leading to diminished fertility	Flow-cytometric analysis	Lliberos *et al.* (2021), Ben Yaakov *et al.* (2023), Isola *et al.* (2024)
Unknown, but the infiltrating CD4+T cells observed with aging are speculated to be Tregs	Hypothesized to infiltrate the ovary to resolve inflammation and restore ovarian integrity	Flow-cytometric analysis	Lliberos *et al.* (2021)
Innate Lymphoid cells: ILC1 ↓; ILC2 and ILC3 unchanged	Unknown	Single-cell RNA-seq; flow cytometric analysis	Ben Yaakov *et al.* (2023), Isola *et al.* (2024)
B cells ↑ or no significant change	Unknown	Single-cell RNA-seq; flow cytometric analysis	Lliberos *et al.* (2021), Ben Yaakov *et al.* (2023), Isola *et al.* (2024)
Natural killer cells ↓ or no significant change	If decreased may lead to a shift toward lymphocyte-rich environment (adaptive immunity)	Single-cell RNA-seq; flow cytometric analysis	Lliberos *et al.* (2021), Ben Yaakov *et al.* (2023), Isola *et al.* (2024)
Dendritic cells: remains unaltered	Unknown	Single cell RNA seq; flow cytometric analysis	Ben Yaakov *et al.* (2023), Isola *et al.* (2024)
Monocytes ↑	Contribute to the percentage of monocyte-derived macrophages in the aged ovary establishing inflammaging; also may represent a compensatory mechanism to address T cell accumulation	Single-cell RNA-seq; flow-cytometric analysis	Zhang *et al.* (2020), Isola *et al.* (2024)
Resident ovarian macrophages ↓; Monocyte-derived macrophages ↑; Alternatively activated macropahges ↑	The elevated ratio of monocyte-derived to tissue resident macrophages may indicate a growing level of monocyte recruitment in the ovarian tissue during aging; a marked decrease in classically activated M1 macrophages may suggest an increased polarization of the ovarian macrophages toward M2 phenotype which is associated with chronic inflammation and tissue fibrosis, a condition present in the aging ovary	Single-cell RNA-seq; flow-cytometric analysis; Immunofluorescence analysis	Zhang *et al.* (2020), Umehara *et al.* (2022), Zhou *et al.* (2023)
Granulocytes			
Neutrophils ↓ or no significant change	Unknown	Single-cell RNA-seq; flow cytometric analysis	Ben Yaakov *et al.* (2023), Zhou *et al.* (2023), Isola *et al.* (2024)
Eosinophils ↑	Contribute to the elevation of IL4 and IL13 signaling leading to increased M2 polarization of ovarian macrophages	Single-cell RNA seq; flow-cytometric analysis	Zhang *et al.* (2020)

CL, corpus luteum; FOXP3, forkhead box P3; Tregs, regulatory T cells.

In addition to macrophages, other immune cell populations populate the ovary, including dendritic cells (DC), natural killer (NK) cells, monocytes, neutrophils, eosinophils, B lymphocytes, T lymphocytes, and innate lymphoid cells (ILCs) (Zhang *et al.* 2020, Lliberos *et al.* 2021, Ben Yaakov *et al.* 2023, Zhou *et al.* 2023, Isola *et al.* 2024). The role that each immune cell population plays in ovarian physiology is not fully elucidated, although a few functional roles have been described (Table 1). For instance, DCs accumulate along with macrophages in periovulatory follicles and contribute to gonadotrophin responsiveness, which is crucial for ovulation and CL formation in both humans and mice (Fainaru *et al.* 2012, Cohen-Fredarow *et al.* 2014). Studies in monkeys indicate that NK cells assist with degrading a regressing CL (Bishop *et al.* 2017). Monocytes are believed to infiltrate the ovary prior to differentiation into macrophages (Zhang *et al.* 2020). Ovarian eosinophils and neutrophils’ functions are unknown but have been speculated to serve a role in CL formation and degradation (Walusimbi & Pate 2013). T lymphocytes, including CD4^+^, CD8^+^, and double-negative (CD4^−^ and CD8^−^), have also been implicated in CL regression in cattle, humans, and mice (Walusimbi & Pate 2013) and CL maintenance during early pregnancy in cattle (Poole & Pate 2012). To our knowledge, the functional significance of ovarian B lymphocytes and ILCs remains unknown and underexplored.

The effect of aging on the abundance of ovarian DCs, NK cells, monocytes, neutrophils, eosinophils, B lymphocytes, T lymphocytes, and ILCs has only recently been explored. DCs are the only ovarian immune cell population that is consistently reported to be unchanged with advancing age in mice (Ben Yaakov *et al.* 2023, Isola *et al.* 2024). Conversely, the number of ovarian NK cells have been shown to decrease or remain unchanged by 9-10 months of age in mice (Lliberos *et al.* 2021, Ben Yaakov *et al.* 2023, Isola *et al.* 2024). Ovarian monocytes and eosinophils have been reported to increase in number with advancing age, but these analyses were performed in 15-month-old mice, therefore it remains unclear if these changes contribute to ovarian insufficiency (Zhang *et al.* 2020). A single report indicated that the amount of ovarian neutrophils decreased by 10 months of age in mice, although the significance of this finding is unclear (Ben Yaakov *et al.* 2023). Conversely, neutrophil abundance in aging human ovaries is unchanged (Zhou *et al.* 2023). Age-related changes in ovarian B lymphocytes have been reported in mice (Lliberos *et al.* 2021), but subsequent studies were unable to confirm this observation (Ben Yaakov *et al.* 2023, Isola *et al.* 2024). Similarly, B lymphocyte abundance is unchanged in human ovaries during aging (Zhou *et al.* 2023). Murine ovarian ILCs were analyzed by two studies and both found that the ILC1 subpopulation was reduced with advancing age, while ILC2 and ILC3 subpopulations were unchanged (Ben Yaakov *et al.* 2023, Isola *et al.* 2024).

To date, four reports have evaluated the effects of aging on T lymphocyte populations in the ovary using single-cell techniques. Unfortunately, the human data lacks sufficient granularity to allow for direct comparisons to the data generated in mice (Zhou *et al.* 2023). In mice, CD4^+^ and CD8^+^ T lymphocytes remain unchanged or modestly increase prior to reproductive failure (Lliberos *et al.* 2021, Ben Yaakov *et al.* 2023, Isola *et al.* 2024). The subpopulation of ovarian T lymphocytes that change most significantly prior to reproductive failure appears to be a broad category of double-negative (CD4^−^ and CD8^−^) T cells (Ben Yaakov *et al.* 2023, Isola *et al.* 2024). These are primarily innate immune cells that can be subdivided into double-negative αβ T cells (DN), natural killer T cells (NKT), mucosal-associated invariant T cells (MAIT), and γδ T cells (γδT) (Pellicci *et al.* 2020, Wu *et al.* 2022). These subtypes can be further stratified into type 1 and type 17 T lymphocytes based on their effector systems. Type 17 T lymphocytes secrete IL-17 and express RAR-related orphan receptor C (Ivanov *et al.* 2006), while type 1 T lymphocytes secrete IFN-γ and express T-box expressed in T cells (T-bet) (Szabo *et al.* 2000). Both type 1 and type 17 T lymphocytes are known to display pro-inflammatory phenotypes (Kumar *et al.* 2021, Nabekura & Shibuya 2021) and have also been linked to inflammaging (Rocamora-Reverte *et al.* 2020). Type 1 NKTs, type 17 DNs, and type 17 γδTs dramatically increase by 9 months of age in mouse ovaries (Isola *et al.* 2024). The functional significance of these observations remains unknown. Interestingly, prior work has established that IL-17 and IFN-γ are linked to the formation of MNGCs (Fais *et al.* 1994, Anderson, 2000, Adamopoulos *et al.* 2015); therefore, the age-related increase in type 1 NKTs, type 17 DNs, and type 17 γδTs may serve a role in the aforementioned accumulation of ovarian MNGCs with aging (Asano 2012, Briley *et al.* 2016, Foley *et al.* 2021, Isola *et al.* 2024). Studies are underway to address this possibility.

### Cellular senescence and ovarian aging

Another factor hypothesized to contribute to age-related ovarian pro-inflammatory stress is the emergence and accumulation of senescent cells. At its core, cellular senescence is a tumor-suppressive mechanism initiated by telomere attrition, mitochondrial dysfunction, and genotoxic, oxidative, and/or mitotic stress (Hernandez-Segura *et al.* 2018, Gorgoulis *et al.* 2019). These factors provoke an irreversible arrest of the cell cycle to prevent the aberrant proliferation of damaged cells. Cells that enter senescence display distinct phenotypic characteristics including increased expression of tumor suppressive genes (Krishnamurthy *et al.* 2004, Sharpless & Sherr 2015, Wiley *et al.* 2017), chromatin remodeling (Narita *et al.* 2003, Swanson *et al.* 2013), changes in DNA methylation (Cruickshanks *et al.* 2013, Cheng *et al.* 2017), and the manifestation of a pro-inflammatory secretome consisting of cytokines, chemokines, and matrix metalloproteinases; termed the senescence-associated secretory phenotype (SASP) (Coppe *et al.* 2008, 2010, Zhu *et al.* 2015). Despite permanent growth arrest, senescent cells are resistant to apoptosis (Yosef *et al.* 2016) and remain metabolically active (Wiley *et al.* 2021). The SASP can adversely affect the local microenvironment by increasing pro-inflammatory responses in neighboring cells and recruiting circulating immune cells (Xu *et al.* 2015, Palmer *et al.* 2019). A major challenge in studying cellular senescence is that a universal marker does not exist, and senescent signatures vary from cell type to cell type.

The magnitude of senescent cell accumulation in the ovary and how cellular senescence promotes ovarian aging and reproductive failure remain unclear. To date, only a few published reports have evaluated markers of cellular senescence in the ovary. The first report showed that p16^INK4A^ increased in the murine ovary with advancing age, and this increase was attenuated by subjecting the mice to calorie restriction (CR) (Krishnamurthy *et al.* 2004). Subsequent studies evaluated a variety of markers linked to cellular senescence. For instance, several reports have established that DNA double-strand breaks (DSBs), a marker commonly associated with cellular senescence (Mah *et al.* 2010), accumulate in aged ovaries from mice, monkeys, and humans (Titus *et al.* 2013, Zhang *et al.* 2015, Saccon *et al.* 2020). Several other studies have shown that ovaries from chronologically aged or obese mice also display increased expression of several senescence-related genes, including *Cdkn2a*, the gene that produces p16^INK4A^, *Cdkn1a*, *Pai1*, *Hmgb1, Igfbp3,* and various SASP factors (Ansere *et al.* 2021, Hense *et al.* 2022, Maruyama *et al.* 2023, Isola *et al.* 2024). *Cdkn1a* has also been reported to be increased in ovaries from middle-aged women when compared to young controls (Zhou *et al.* 2023). Lipofuscin aggresomes, which are considered a marker of cellular senescence (Evangelou & Gorgoulis 2017), have also been reported to accumulate significantly in the aged mouse ovary (Urzua *et al.* 2018, Ansere *et al.* 2021, Hense *et al.* 2022, Isola *et al.* 2024). Lastly, β-galactosidase positivity, a classical marker of senescent cells (Dimri *et al.* 1995), increased with age in the mouse ovary (Maruyama *et al.* 2023).

Interestingly, many of the previously mentioned studies were performed in mice that were in the latter stages of their reproductive window, but prior to reproductive failure. Therefore, it is plausible that many of these senescence-related markers are at least associated with, if not causally implicated, in fertility declines. However, a panel of specific markers for ovarian senescent cells is yet to be developed, and the detection of one or several of the aforementioned senescent cell-related signatures does not unequivocally indicate cellular senescence (Sharpless & Sherr 2015, Hernandez-Segura *et al.* 2018). For example, the severity of DNA DSBs determines whether the cell repairs the breaks, undergoes apoptosis, or becomes senescent (White & Vijg 2016) and gene expression changes often ascribed to senescent cells are not exclusive to this cellular state (Salmonowicz & Passos 2017), although *Cdkn2a* and *Cdkn1a* remain fairly predictive (Gorgoulis *et al.* 2019). β-galactosidase positivity also has major limitations because it can arise in quiescent cells (Cristofalo 2005). Despite the recent rise in popularity of lipofuscin positivity as a hallmark of senescent cells, a recent report demonstrated that ovarian lipofuscin strongly associates with MNGCs (Isola *et al.* 2024), which suggests lipofuscin may not be a good marker of cellular senescence in the ovary, or that MNGCs are actually a collection of fused senescent cells. Additional studies will be needed to unravel this speculation. Additionally, senescence-associated genes in the ovary are predominantly expressed by immune cells and did not change with age. Therefore, increased ‘cellular senescence’ during ovarian aging may actually reflect increased immune cell abundance (Isola *et al.* 2024). However, it is noteworthy that *Cdkn1a* expression increased in type 17 T lymphocytes with advancing age, representing a small subpopulation of cells that likely enter cellular senescence in the ovary. Additional studies will be needed to create a definitive panel of senescence biomarkers that are specific to the ovary, and these efforts are currently underway in mice and humans through the Cellular Senescence Network (SenNet Consortium 2022).

### Interventional strategy for curtailing ovarian pro-inflammatory processes

Several prior studies have evaluated the effects of dietary or pharmacological interventions on ovarian aging. Many of these strategies have proven beneficial in preserving ovarian reserve and fertility with advancing age. For instance, 4 to 6 months of CR starting early in the reproductive window attenuated the loss of PFs by nearly half in mice (Selesniemi *et al.* 2008, Garcia *et al.* 2019, Isola *et al.* 2022), and this translated to improvements in fertility after refeeding had been established (Selesniemi *et al.* 2008, Zhou *et al.* 2014, Isola *et al.* 2022). Similar results have been observed with metformin and rapamycin treatment. Metformin attenuated age-related loss of PFs during the reproductive window (Qin *et al.* 2019) and diminished irregular estrous cycles in mice (Anisimov *et al.* 2011, Qin *et al.* 2019). Short-term (2 weeks) and long-term (6 months) rapamycin administration also increased PF numbers in mice (Dou *et al.* 2017, Garcia *et al.* 2019), which is associated with improved fertility rates later in life (Dou *et al.* 2017). Antioxidants and anti-inflammatory drugs have also shown promise for improving ovarian reserve and fertility. Both resveratrol and MCC950, an inhibitor of the NLRP3 inflammasome, delayed age-related PF exhaustion and improved measures of fertility (Liu *et al.* 2013, Navarro-Pando *et al.* 2021, Gou *et al.* 2023). In recent years, NAD^+^ precursors have also garnered interest for their potential to improve reproductive outcomes, by improving ovarian reserve and fertility measures in mice (Bertoldo *et al.* 2020, Yang *et al.* 2020, Huang *et al.* 2022). Collectively, the data outlined above indicate that ovarian aging can be manipulated through interventional approaches. However, it remains unclear if the aforementioned improvements in reproductive outcomes are occurring in response to direct effects in the ovary or secondarily to systemic benefits.

To date, only a few studies have evaluated the effects of dietary or pharmacological interventions on age-related changes in ovarian inflammatory phenotypes. With regard to CR, one report suggested that it did not elicit beneficial effects on inflammation in the ovary (Sharov *et al.* 2008). The effects of metformin on ovarian inflammation are also only sparingly explored. For instance, treatment with metformin during the reproductive window prevented age-related increases in the senescence marker p16^INK4A^; however, no other measure of inflammation or cellular senescence were evaluated (Qin *et al.* 2019). Notably, administration of metformin following reproductive failure suppressed ovarian pro-inflammatory responses, which was mirrored by changes in the immune cell landscape (Landry *et al.* 2022). This aligns with a human study that found metformin can reverse the age-related rise in alternatively activated macrophages within the ovary of postmenopausal women (McCloskey *et al.* 2020). However, it remains unclear if metformin would modulate these parameters if provided during the reproductive window. Short-term rapamycin treatment reduced ovarian pro-inflammatory cytokines, but this effect was not sustained over time (Dou *et al.* 2017). The most extensive characterization of an interventional compound found to modulate ovarian inflammation was done with the small molecule BGP-15, a hydroximic acid derivative (Wachal *et al.* 2020). This report demonstrated that BGP-15 reduced age-related macrophage accumulation and cytokine production within the ovary (Umehara *et al.* 2022). Importantly, these benefits coincided with declines in ovarian fibrosis, indicating that several hallmarks of ovarian aging can be attenuated simultaneously by a single drug (Umehara *et al.* 2022). The evaluation of additional dietary and pharmacological interventions on age-related ovarian outcomes is needed, particularly those that have curtailed pro-inflammatory mechanisms in other organ systems. The use of ovarian cell type-specific transgenic mice in combination with interventional strategies could also aid in determining if ovarian benefits occur through actions on follicular or stromal cells.

## Conclusion and future directions

**Figure 1:**
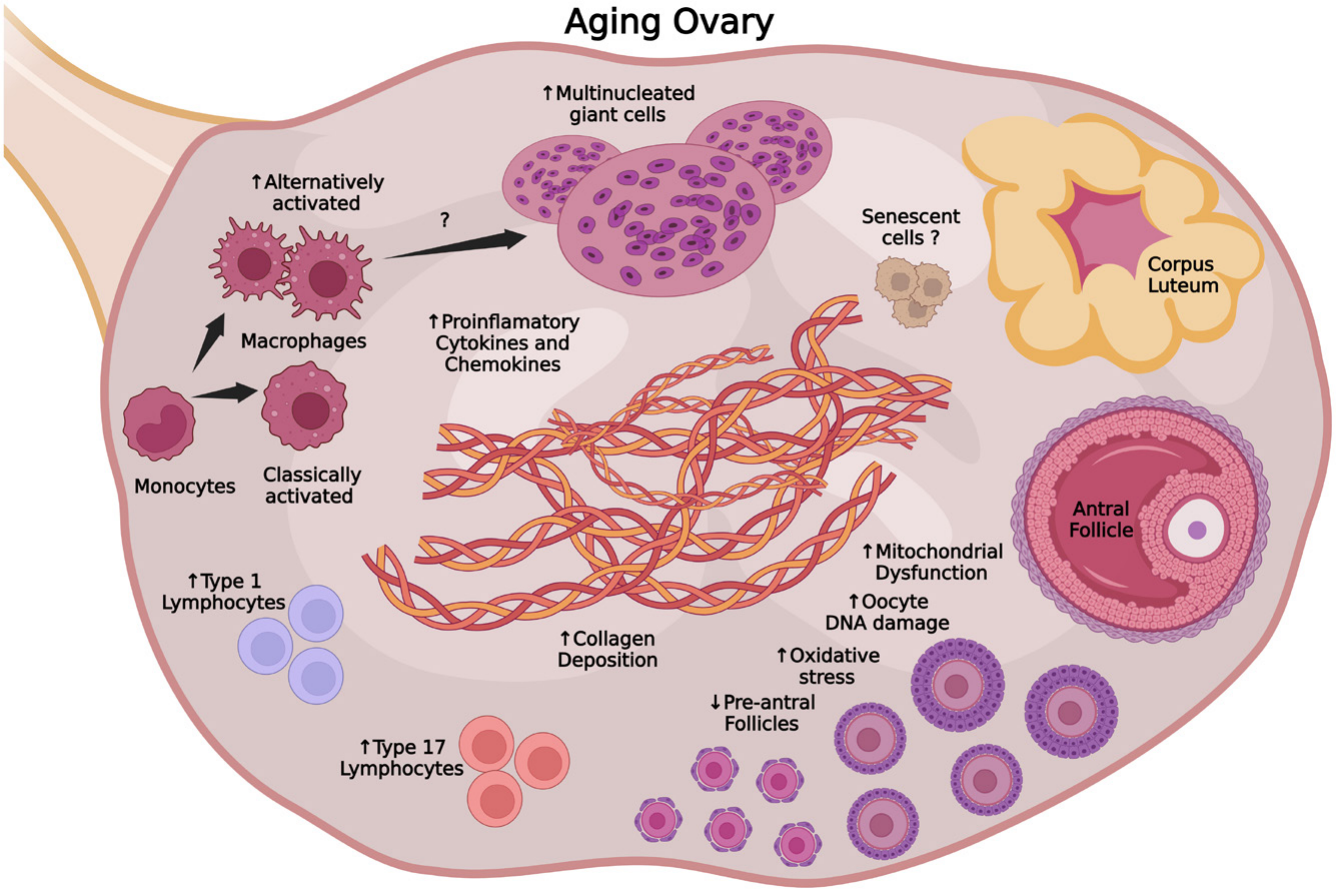
Schema of age-related changes in ovarian cellularity and biomarkers of functional declines. Ovarian aging is characterized by a decline in follicular reserve and an increase in pro-inflammatory mediators, collagen deposition, oxidative stress, mitochondrial dysfunction, and accumulation of multinucleated giant cells. Some reports suggest that senescent cells accumulate in the aged ovary. Recent findings indicate that these hallmarks of ovarian aging correspond to changes in immune cellularity. The ratio of alternatively activated to classically activated macrophages increases with age and type 1 and type 17 T lymphocytes accumulate in parallel. It remains unclear if the changes in immune cell populations underlie the aforementioned ovarian aging hallmarks. Therefore, additional studies are needed to disentangle this potential relationship. This figure was created with Biorender.com.

Recent data indicate that changes in ovarian inflammation and immune cell abundance are associated with ovarian aging hallmarks (e.g. loss of PFs, fibrosis, MNGC accumulation) (Fig. 1) and may be causally implicated in ovarian functional declines. However, more research needs to be done to definitively determine the relationships between inflammation, immune cell abundance, and ovarian failure. No studies to date have directly ablated pro-inflammatory mediators in the ovary, and studies employing global knockouts make it difficult to disentangle how systemic and local changes in pro-inflammatory mechanisms contribute to ovarian aging. Furthermore, the precise roles that each immune cell population plays in modulating ovarian function and pathology remain unresolved. For example, additional studies are needed to determine how type 1 and type 17 T lymphocytes are involved in ovarian aging. The functional importance of MNGCs in the ovary also remains unclear, as well as the mechanisms that promote their formation with advancing age. Although MNGCs are believed to be associated with pro-inflammatory signals, no assessments of their secretomes have been performed. Additional studies addressing the effects of interventions in ovarian aging should also assess pro-inflammatory markers and changes in immune cellularity, as inflammation and immune response appear to be intimately involved in ovarian aging. Despite the challenges associated with obtaining healthy samples across the life span, more investigation of human ovaries is needed in order to confirm that findings in mice are translatable to humans.

## Declaration of interest

The authors declare that there is no conflict of interest that could be perceived as prejudicing the impartiality of this review.

## Funding

This work was supported by the National Institutes of Healthhttp://dx.doi.org/10.13039/100000002 (R01 AG069742 to MBS), Global Consortium for Reproductive Longevity and Equality (GCRLE-4501 to MBS and GCRLE-0523 to SRO), and Presbyterian Health Foundationhttp://dx.doi.org/10.13039/501100000724 (Pilot Research Funding to MBS).

## Author contribution statement

JVVI and MBS conceived the review. JVVI, JDH, CAPO, SB, JAI, SRO, AS, and MBS evaluated the available literature and drafted sections within the manuscript. JVVI and MBS revised the manuscript and created the final draft. All authors approved the final draft of the manuscript.
